# The larva of *Eustra* (Coleoptera, Paussinae, Ozaenini):a facultative associate of ants

**DOI:** 10.3897/zookeys.90.1136

**Published:** 2011-04-14

**Authors:** Wendy Moore, Xiao-bin Song, Andrea Di Giulio

**Affiliations:** 1 *Department of Entomology, University of Arizona, Tucson, Arizona 85721-0036, USA*; 2 *Shanghai, P.R. China*; 3 *Dipartimento di Biologia Ambientale, Università “Roma Tre” Viale G. Marconi 446, I-00146 Rome, Italy*

**Keywords:** flanged bombardier beetles, myrmecophily, systematics, larvae, Southeast Asia

## Abstract

Larvae of the ground beetle genus Eustra Schmidt-Goebel are described and illustrated for the first time and some biological notes are reported. One specimen of an unknown Eustra species was collected while excavating a nest of the ant Pachycondyla javana Mayr, in Taiwan, which is the first report of a paussine associated with a member of the ant subfamily Ponerinae. Several larvae and adults of a second species, Eustra chinensis Bänninger, were collected in Shanghai under bark with no association with ants. First instar larvae of the latter species were also reared in the lab. The occurrence of larvae of the genus Eustra both inside and outside ant nests, together with a report of adults collected inside a nest in Taiwan, suggests that members of this genus may be facultative predators or facultative symbionts of ants, an attribute that has never been reported for this genus. The larvae of Eustra show several unique features, including a peculiar bidentate mandibular apex, an extremely long galea, one of two tarsal claws greatly reduced, abdominal setae (including those of terminal disk) elongate and clavate at apex, urogomphi wide and flattened, and inflated sensilla S-I. Larvae were studied by both optical and scanning electron microscopy, their morphological features are compared with those of other described Paussinae larvae, and their potential phylogenetic and functional significance are discussed.

## Introduction

Eustra Schmidt-Goebel is an ozaenine genus (Carabidae: Paussinae) containing twenty-two species (reviewed by Deuve 2001). Adults in this Southeast Asian genus have the smallest body size of all members of the subfamily Paussinae. Many Eustra species live in caves and exhibit typical structural adaptations to a troglobitic life, including loss of pigmentation, loss of eyes, and long, delicate appendages. Other species are not cave-dwelling, but rather they have been collected in microhabitats typical of other ozaenines including under rocks and under bark. Recently larvae of two species of Eustra were collected in the field. Both adults and larvae of Eustra chinensis Bänninger were collected in Shanghai while they were hibernating in rotting wood and a single larva of an unidentified species of Eustra was collected during the excavation of nests of the ant Pachycondyla javana Mayr in Taiwan, suggesting for the first time that at least some species of Eustra are facultatively associated with ants (Moore 2006).

Many different animals, especially arthropods, profit from a facultative or obligate association with ants (myrmecophily), bypassing the behavioral and chemical defenses of the hosts and adapting to the peculiar environmental conditions of the nests. Since myrmecophiles are rare and live in concealed environments, our knowledge of their behavior is sparse, and most of the information we do have has been inferred from structural features of adults and larvae (Di Giulio and Moore 2004; Di Giulio et al. 2011). The Paussinae, commonly known as flanged bombardier beetles, are a good model taxon to study the evolution of myrmecophily in beetles, since members of this ground beetle subfamily have different degrees of associations with ants, ranging from apparently none to obligate myrmecophiles (see Geiselhardt et al. 2007 and references therein). As far as we know all members of the tribes Protopaussini and Paussini are myrmecophilous, at least during the larval stage, and their associations with ants have either been directly observed in the field or deduced from their remarkable structural adaptations. Most of these species associate with members of the ant subfamilies Formicinae and Myrmicinae.

In general, very little is known about the behaviour of the ozaenines (Di Giulio and Vigna Taglianti 2001; Moore and Di Giulio 2006; Moore 2008). Like most ground beetles, they are usually found under stones, bark, and rotting wood and they are night-active predators on other arthropods. Ozaenine larvae are known for only nine species in four genera (Itamus, Sphaerostylus, Pachyteles and Physea) (see Di Giulio and Moore 2004). They all have a terminal disk composed of modified abdominal tergites and urogomphi, which is a synapomorphy for the subfamily (Bousquet 1986). Unlike the physogastric myrmecophilous larvae of the tribe Paussini which use their round terminal disk as a glandular symphilous organ (Oberprieler 1985; Bousquet 1986; Luna de Carvalho 1989; Di Giulio and Moore 2004; Di Giulio et al. 2011), free-living larvae of Metriini, Mystropomini and Ozaenini use their terminal disk as a door to close the galleries they construct in rotten wood, humid earth or sandy riverbanks, and they use the moveable components of the terminal disk to trap their prey (Costa et al. 1988; Di Giulio and Vigna Taglianti 2001), seizing them with their sharp mandibles through a backward spring-like movement. This specialized feeding strategy allows these delicate larvae to feed on fast moving invertebrates and to occasionally feed on ants. It is likely that many non-myrmecophilous members of the subfamily Paussinae facultatively feed on ants, as has been demonstrated in the tribe Metriini (Moore and Di Giulio 2008). We hypothesize that myrmecophagy may be a preadaptation for myrmecophily.

Members of the ozaenine genus Physea Brullé are known to live inside the nests of the Neotropical leafcutting ants, Atta, and both larvae and adults have structural adaptations for this lifestyle (Eidmann 1937, Di Giulio et al. 2003). Recently adults of other ozaenine species have been found inside Atta nests including adults of Tachypeles moretianus Deuve and Serratozaena paraphysea Deuve (Moore 2008). Based on structural features of adults, myrmecophily has also been hypothesized for the Southeast Asian species Dhanya mulu Stork (Stork 1985), and the South and Central American genera Ozaena Olivier and Platycerozaena Bänninger (Ball and McCleve 1990). Larval specimens of the Malagasy species Sphaerostylus goryi (Laporte de Castelnau) were recently collected both in rotten wood with ants and in leaf litter without apparent association with ants (WM, personal observations). The discovery of an Eustra larva inside a Pachycondyla javana nest in Taiwan is the only report of a paussine associated with a member of the ant subfamily Ponerinae (Moore 2006). That Eustra larvae have been collected both inside and outside of ant nests suggests the possibility that they are at least facultatively associated with ants. In addition, a report of adults of Eustra sp. collected inside an ant nest in Taiwan was posted on the Internet (http://nc.kl.edu.tw/bbs/showthread.php?t=653&page=9). These findings suggest that more ozaenine taxa may be facultative or obligate myrmecophiles, even those without evident structural adaptations, and they suggest that myrmecophily has evolved multiple times during the evolution of in Paussinae (Moore 2006; Moore et al. 2010).

In this paper we: (1) present biological information about the habitats and behaviors of Eustra chinensis and the Eustra species from Taiwan observed in nature and in captivity; (2) describe and illustrate these larvae; (3) discuss the functional significance of several unique characteristics of the genus; and (4) compare them to other described paussine larvae.

## Methods

### Material described

(1) Eustra chinensis Bänninger, 1949. Twenty-five adults and several larvae were collected in Shanghai on February 9, 2009 and April 6, 2008. They were found hibernating together in the soft, rotten wood of bristly locust (Robinia hispida Linnaeus,1767) and weeping willow (Salix babylonica Linnaeus,1753). Adults and larvae can be found in Shanghai throughout the year. They overwinter as both adults and larvae (all larval instars), from November to April. Presumably, while these larvae are overwintering they do not feed. In captivity, a third instar larva overwintered without food for more than six months. During this time they did not close the opening of their galleries with their terminal disks, as they do to facilitate feeding during the spring and summer (as described for other ozaenine larvae, see Costa et al. 1988; Di Giulio and Vigna Taglianti 2001; Moore and Di Giulio 2006).

(2) Eustra sp. A single third instar larva was collected in northern Taiwan (Shan-shya [sic]) by Gustav Tzh-wei Chen on 9.IX.2003 while excavating a nest of Pachycondyla javana Mayr (Hymenoptera, Formicidae, Ponerinae). The specimen was identified as belonging to the genus Eustra, by a phylogenetic analysis of molecular sequence data obtained from this specimen and from sixty other members of the subfamily Paussinae, including other members of the genus Eustra.

### Rearing conditions

Larvae of Eustra chinensis were reared in captivity, where ambient conditions (*e.g.*, temperature, light and humidity) were similar to natural conditions outdoors. Five larvae of each instar were reared in 2 ml centrifuge tubes. Other larvae were reared in a plastic box (18 cm × 11 cm × 12 cm) with the field-collected rotten wood. All larvae were fed springtails once a month.

### Morphological analysis

Prior to preparing them for microscopy, larvae were drawn by a stereomicroscope Olympus SZX16 equipped with drawing tube. One specimen of each instar of Eustra chinensis, and the single specimen from Taiwan were rehydrated, cleared in 10% KOH, transferred in hot lactic acid, dehydrated through a series of EtOH baths of increasing concentration (10-20-50-70-90-95-100%), left overnight in a clove oil bath, and mounted on slides with Canada balsam. These specimens were studied and illustrated by using a light microscope Olympus BX51 equipped with drawing tube. Another first instar specimen was dehydrated through a series of EtOH baths of increasing concentration (70-80-90-95-100%), critical point dried (Bal-Tec CPD 030), mounted on a stub (by using self adhesive carbon disks), sputtered with gold (Emitech k550 sputter coater), and observed with Philips XL30 scanning electron microscope and FEI Dualbeam FIB/SEM Helios Nanolab (L.I.M.E. laboratory, University ‘Roma Tre’, Rome). In this paper, the general terminology of larval structures follows Lawrence (1991). Notation of primary setae and pores follows the system of Bousquet and Goulet (1984), modified for Metrius contractus (Bousquet 1986). Because some of the sensilla on the abdomen and terminal disk of Eustra are homologous to those recognized in Metrius contractus (sensilla S-I to S-V) (Bousquet 1986), Pachyteles spp. (sensilla S-I to S-VII) (Di Giulio et al. 2000), and Arthropterus sp. (sensilla S-I to S-VIII) (Di Giulio and Moore 2004), we adopt here the same nomenclature used by these authors. Notation of microsculpture follows Harris (1979). An asterisk (*) following a coded seta indicates that the homology between the structure on the Eustra larvae and the corresponding code is questionable.

## Results

### Eustra larval morphology

**Generic diagnosis.** Body length very small as compared with other Paussinae (1.75mm, first instars); antenna 3-jointed (II+III fused); mandible apically bidentate, with sub-basal retinaculum, ental margin of retinaculum with additional small sub-basal tooth; galea extremely long and apically sharp, distinctly longer than maxillary palp and lacinia; maxillary palpomere 3-jointed (II+III fused); claws of very different size, smaller claw obsolescent; hypopleurite VI with ventrolateral, elongate digitiform protuberance, tipped by strong spine-like seta; most sternal and pleural setae of the abdomen elongate and clavate at apex; lateral plates of terminal disk thin and wing-like, pointed at apex, with dorsal margin straight and ventral margin curved; urogomphi flattened, wider and longer than dorsal plates, composed by 7 short triangular lobes, acute at apex, separated by V-shaped incisures of different depths; lobe X present between C and E2; lobe E1 divided into E1a and E1b; peculiar mushroom-like inflated sensilla S-I of different length present on surface of plates and urogomphi; sensilla S-II of two different types, alternate on dorsal plates and urogomphi: (1) very long and stick-like, pointed at apex; (2) short and clavate at tip; terminal disk covered with peculiar hairy microsculpture.

### Eustrachinensis first instar larva

**Habitus and coloration.** Body soft, whitish, weakly sclerotized, not physogastric; abdomen flattened, bellows-like, contracted dorsally elevating the large terminal disk; terminal disk, cephalic capsule and mouthparts well sclerotized, yellowish to light brown; mandibles, laciniae, anterior margin of frontal sclerite, egg-bursters and claws thickly sclerotized and reddish brown.

**Figure 1.**
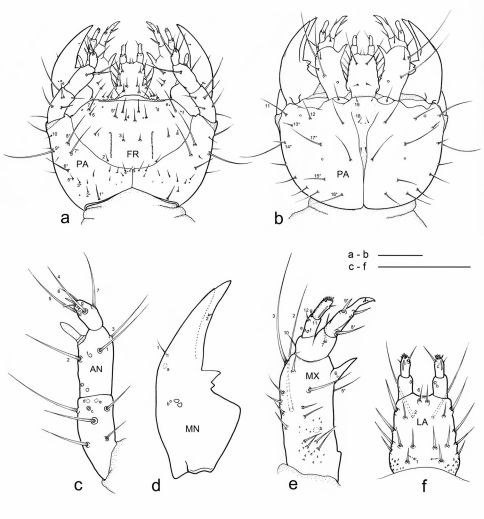
Eustra chinensis first instar larva: line drawings of **a** head, dorsal view **b** head, ventral view **c** left antenna, dorsal view **d** left mandible, dorsal view **e** left maxilla, dorsal view **f** labium, dorsal view. Scale bars: 0.1 mm.

**Figure 2.**
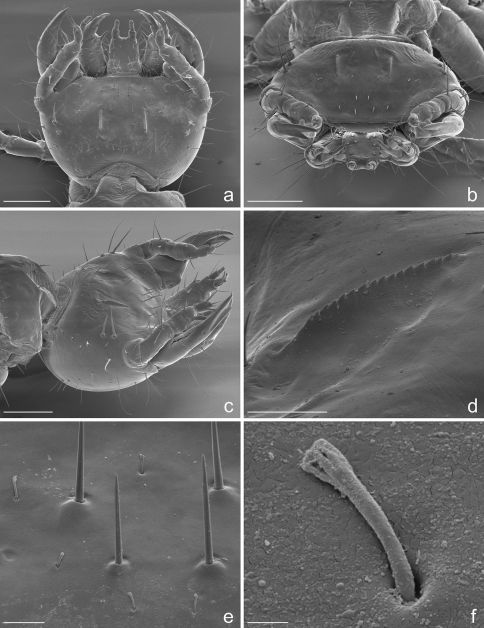
Eustra chinensis first instar larva: SEM micrographs of **a** head, dorsal view **b** head, frontal view **c** head, dorsolateral oblique view **d** left egg-burster, right dorsolateral oblique view **e** basal setae of frontoclypeolabrale, with filiform sensilla, dorsal view **f** close-up of a filiform sensillum of frontoclypeolabrale. Scale bars: **a**, **b**, **c** = 100 µm; **d** = 20 µm; **e** = 10 µm; **f** = 1 µm.

**Figure 3.**
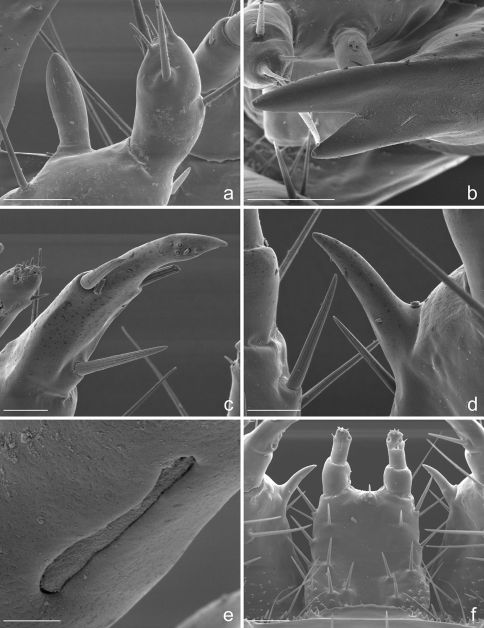
Eustra chinensis first instar larva: SEM micrographs of **a** apex of left antenna, dorsal view **b** apex of left mandible, lateral view **c** apex of left galea, dorsal view **d** right lacinia, dorsal view **e** digitiform sensillum of maxillary palpomere IV, lateral view **f** labium, dorsal view. Scale bars: **a** = 20 µm; **b**, **f** = 25 µm; **c**, **d** = 10 µm; **e** = 2 µm.

**Microsculpture.** Cephalic capsule, mouthparts, thoracic tergites and legs smooth, without or with only sparse, pointed microsculpture (Figs 2, 5a, 5b); anterior margin of frontoclypeolabrale to adnasalia strongly denticulate at sides of median prominence, resulting in a serrate anterior edge (Figs 1a, 2a); anterior frontal keel smooth; basal third of prementum (Figs 1f, 2a, 3f) and stipes (Figs 1e, 2a, 3f) with pointed microsculpture on dorsal surface; membranous areas of the body and sclerites of the abdomen rugulose to rugose (Figs 5a, 5c-f), with pointed or multi-pointed sculpticells, sparse near the setae, longer on epipleurites I-VII; dorsal surface of dorsal plates, basal part of lateral plates and ventral surface of urogomphi covered by transverse rows of 2-6 spines (2-3 µm long), regularly spaced every 3-6 µm (Fig. 7d); surface of terminal disk (Fig. 7) thickly covered by a peculiar hairy microsculpture; pygidium, with pointed to multi-pointed microsculpture.

**Figure 4.**
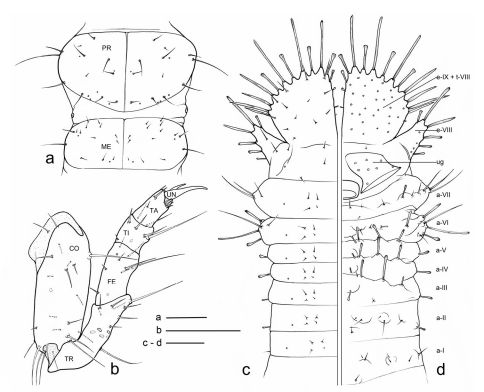
Eustra chinensis first instar larva: line drawings of **a** pro- and meso-notum, dorsal view **b** left foreleg, anterior view **c** right side of abdomen, dorsal view **d** right side of abdomen, ventral view (right urogomphus not drawn). a- = abdominal segment; e- = epipleurite; t- = tergum; Scale bars: 0.1 mm.

**Figure 5.**
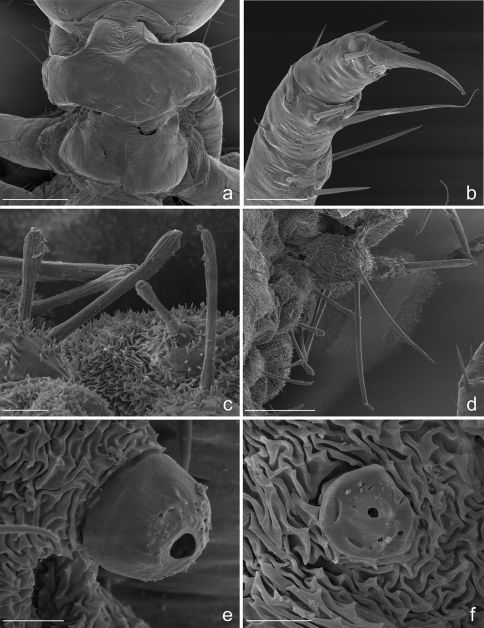
Eustra chinensis first instar larva: SEM micrographs of **a** pro- and meso-notum, dorsal view **b** apex of left hind-leg, anterior view **c** abdominal sensilla on left pleurae, dorsal view **d** abdominal sensilla on left pleurae, dorsal view **e** right metathoracic spiracle **f** abdominal spiracle I. Scale bars: **a** = 100 µm; **b** = 30 µm; **c** = 10 µm; **d** = 50 µm; **e** = 5 µm; **f** = 4 µm.

**Figure 6.**
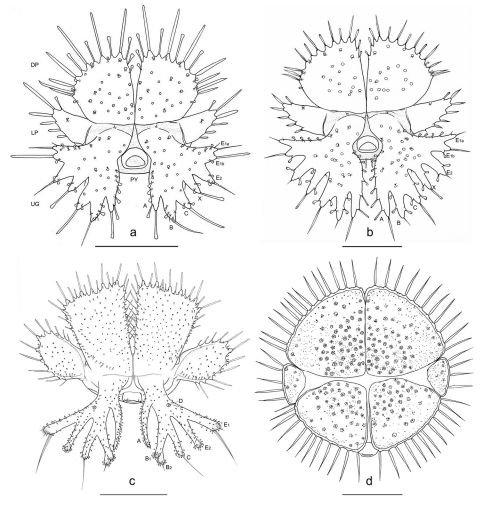
Terminal disks of: **a** Eustra chinensis first instar **b** Eustra sp. Taiwanthird instar **c** Goniotropis kuntzeni first instar **d** Paussus favieri first instar. DP = dorsal plate; LP = lateral plate; UG = urogomphus; PY = pygidium. Scale bars: a = 0.25 mm; b= 0.5 mm.

**Figure 7.**
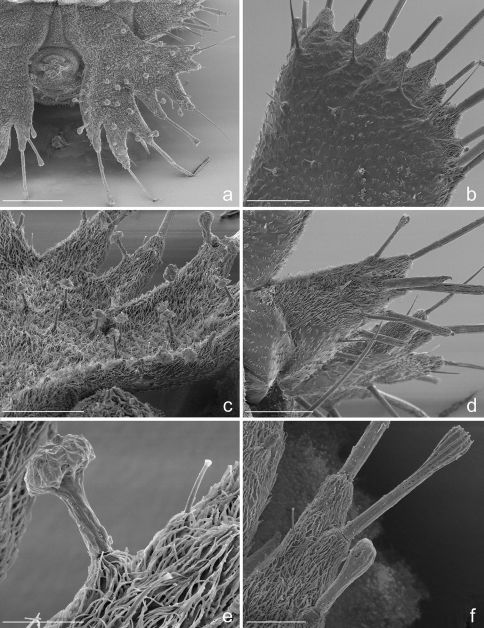
Eustra chinensis first instar larva: SEM micrographs of **a** right urogomphus, dorsal view **b** left dorsal plate, dorsal view **c** right urogomphus, left dorso-lateral view **d** left lateral plate, dorsal view **e** sensillum S-I on X lobe, lateral view **f** right urogomphus, lobe A, dorso-lateral view. Scale bars: **a** = 100 µm; **b**, **c**, **d** = 50 µm; **e** = 10 µm; **f** = 20 µm.

**Chaetotaxy.** Frontoclypeolabrum (Figs 1a, 2a-c) without additional setae; FR1-4,6-9 easily distinguished (Fig. 1a); medial prominence of frontoclypeolabrum with 2 minute spine-like setae on dorsal surface (Fig. 2b); FRb absent; several minute  filiform sensilla (Fig. 2b) expanded at apex present on anterior part of frontale (Figs 2e-f). Parietale (Figs 1a, 2c) with several small additional setae irregularly positioned mesodorsally, and longer additional setae placed ventrally; some setae of parietale possibly homologous to the ancestral pattern are tentatively assigned in Fig. 1a. Antennomere I (Figs 1c, 2c) with 5 dorsolateral additional setae; ANa,b absent; III with AN1 and ANf displaced apically (Fig. 1c). Mandibles (Fig. 1d) with two large additional pores mesodorsally. Setal group gMX on stipes composed of about 10 setae (Fig. 1e); MX6 very small, dorsal and subbasal on lacinia; galeomere II with one additional seta on ental side and a subapical, dorsal sensorial area (composed of 3 dome-like and 1 longer medial sensilla) (Fig. 3c); maxillary palpomere IV with 1 small additional seta on ental side, 2 longitudinal subapical digitiform sensilla (Fig. 3e) and apical sensorial area composed of several papillae. Prementum (Figs 1f, 3f) with about 10 additional setae on lateral and dorsal surface, LA3, 4, 5 not clearly identifiable; seta LA1 close to the midline; LAa absent, LAc subapical; labial palpomere II with 2 additional setae, 1 dorsal, medially directed and 1 small ventrolateral, 2 longitudinal subapical digitiform sensilla and apical sensorial area composed of several papillae. Pro-, meso- and metanotum (Figs 4a, 5a) with about 25 setae each (identification not possible). Coxa with about 20 setae; trochanter with spiniform setae present mostly on ventral side, including a long TR4; TR8 about as long as TR4 but thinner and more flexible. Meso- and metasternum with MS4 long. Abdominal tergites I-VII (Fig. 4c) with 4 setae on each side. Tergal side of dorsal and lateral plates of terminal disk (Figs 4c, 7b) with stiff pointed setae (sensilla S-VII) of various sizes, with cylindrical bases protruding from the plates: about 14 on each dorsal plate (epipleurite IX + tergite VIII) and about 3 on each lateral plate (epipleurite VIII); distal margin of each dorsal plate with about 12 elongated, straight and deeply corrugated sensilla S-II, of two different sizes and shapes (Fig. 6a) alternately placed: type 1 extremely long (about double than type 2), stick-like, with sharp tip; type 2 thinner than 1 and distinctly clavate at apex; inner edge of each dorsal plate (Fig. 6a) with 2-3 S-II type 2 obliquely directed, increasing in size from base to apex; margin of each lateral plate with 8 sensilla S-II, 5 of type 1; caudal side of the terminal disk with numerous sensilla S-I (Figs 4d, 6a) sparsely distributed: 25-30 S-I on each dorsal plate and about 1-4 on each lateral plate. Epipleurites (Figs. 4c-d, 5c-d) of abdominal segment I without setae, II-V with one elongate sensillum S-II (type 2) each, VI-VII with several setae and S-II type 2. Sternal area of segment I with small simple setae, II-VI with elongate sensilla S-II type 2, VII with simple elongate setae (except for one, see Fig. 4d). Urogomphi (Figs 6a, 7a, c) with many S-I (about 40), mainly on dorsal surface and at margins of branches; branches A, C, X and E1b with S-II type 2 (Fig. 7a), B, E2, E1a with apical long S-II type 1 (Fig. 7a, f). Pygidium without setae (Fig. 7a).

**Head.** Cephalic capsule (Figs 1a-b, 2a-c) strongly transverse (width/length ratio = 1.86), hyperprognathous, rounded laterally, regularly tapered at basal half into a distinct neck; maximum width at antennal insertions about twice as wide as occipital foramen. Frontoclypeolabrum (Figs 1a, 2a,c) strongly transverse (width/length ratio = 1.64), with surface medially convex and anterolaterally concave; basal stem of epicranial suture short, anterior frontal arms only slightly sinuate; egg-bursters (Fig. 2d) composed of two longitudinal, multispinulate carinae, each consisting of about 20 forwardly directed teeth; carinae parallel, about one third the length of frontoclypeolabrum, widely separated, placed between FR1 and FR3. Anterior margin of frontoclypeolabrum (Figs 1a, 2a-b) double-edged: dorsal edge smooth, slightly convex, forming a transverse keel (see Di Giulio et al. 2003 and Di Giulio and Moore, 2004 for a discussion on the homology) extended to the adnasalia, ventral edge strongly serrate laterally, medially produced into a wide subrectangular labral tooth (labral spine *sensu* Beutel 1992); adnasalia slightly rounded and slightly protruding. Parietale (Figs 1a-b, 2c) without stemmata; ocular and cervical grooves absent; ventral walls of parietale medially fused into a complete gular suture (Fig. 1b). Antennae (Figs 1a, 2a) strongly directed mesad, 3-jointed; antennomere I (first joint) about as long as II+III (second joint) and twice as long as IV (third joint); sensorial appendage (Figs 1c, 3a) elongate, bullet-like, slightly shorter than IV, laterally positioned at apex of III. Mandibles (Figs 1d, 3b) stout, sickle-shaped, about 2 times as long as wide at base, regularly curved along lateral margin; apex sharply bidentate, lower tooth smaller than upper and distinctly divergent; penicillus absent; terebra slightly convex beyond retinaculum, then concave to apex; retinaculum small sub-triangular, sub-basal in position, postero-medially directed; anterior margin rounded and convex, posterior margin straight, with sub-basal tooth. Maxilla (Figs 1e, 3c) with undivided cardo, subrectangular stipes, 3-jointed palp, 2-jointed galea and 1-jointed lacinia. Stipes distinctly curved inward, about 3 times as long as wide at base; small tooth-like protuberance present subbasally on the ental side of stipes. Maxillary palpi short; palpomere I wide and subconical, medially fused with basal galeomere; II fused with III forming a unique second joint; IV (third joint) elongate, digitiform, about as long as II+III combined. Galea very long, distinctly longer than palpus; galeomere I short, stout; II elongate, hook-like, apically sharp and inward directed, about two times as long as I; galea+palpus distinctly up-curved. Lacinia (Figs 1e, 3d) very short and slender, almost straight, strongly sclerotized, basally fused with stipe. Labium (Figs 1f, 3f) with slightly sclerotized prementum and 2-jointed palpi; prementum subrectangular, distinctly decreasing in width from base to apex; small setal notches present on dorsal surface and sides; ligula very short, dome-like, sub-dorsal; labial palpomere I cylindrical, slightly shorter and wider than II; II digitiform, slightly compressed apically.

**Thorax.** Tergites (Figs 4a, 5a) scarcely sclerotized, sternum not sclerotized. Pronotum wider than meso- and metanotum, transverse, about two times wider than long. Meso- and metanotum widely transverse, about two and a half times wider than long; longitudinal ecdysial line well marked on pro- and mesonotum, less evident, but present, on metanotum.

**Spiracles.** Thoracic and abdominal spiracles (Figs 5e, f) annular-uniphorous; mesothoracic spiracles dome-like, anterolateral on mesopleura, more than twice the size of the abdominal spiracle I. Abdominal spiracles rounded, plate-like, not protruding (Fig. 5f), placed dorsolaterally between tergites and epipleurites.

**Legs.** Legs well developed, 5-jointed (Fig. 4b), forelegs slightly shorter than others, mid and hind legs subequal. Coxa cylindrical, very long, about as long as trochanter and femur combined; trochanter elongate, obliquely truncate and fused apically to femur, about as long as femur and tibia combined; femur about as long as tibia and tarsus combined; tibia very short, cylindrical, slightly shorter than tarsus; tarsus more slender than tibia, conical, tapered from base to apex, with 2 sharp unequal claws (Fig. 5b): anterior claw elongate and strong, slightly longer than tarsus, apically curved; posterior claw very small and somewhat obsolescent.

**Abdomen.** Abdominal segments I-VII (Figs 4c, d) not sclerotized, bellows-like, usually up-curved, keeping the abdominal apex in an elevated position. Abdominal sclerites barely discernable, recognised by reduction of multipointed microsculpture around setae or sensilla S-II; segments progressively wider from I to VIII. Each segment dorsally flattened, with swollen, setiferous pleural and sternal areas. Hypopleurites setiferous, slightly protruding; hypopleurite VI with ventrolateral, elongate digitiform protuberance, tipped by strong spine-like seta. Epipleurites conical, distinctly protruding, gradually more developed from segment I to VIII; epipleurites of segment VIII (Figs 4c-d, 6a, 7d) flattened and enlarged into two sclerotized lateral plates, smaller than the dorsal plates; lateral plates slender, triangular, about two times longer than wide at base; epipleurites of segment IX greatly enlarged and fused with tergum of segment VIII into two rectangular, sclerotized plates (dorsal plates), slightly enlarged from base to apex and widely separated in the middle by a deep V-shaped notch (Figs 4c-d, 6a); lateral plates widely separated from dorsal plates; lateral plates, dorsal plates and urogomphi forming a terminal disk articulated at base by membranes, dorsal and lateral plates move against urogomphi. Urogomphi (Figs 6a, 7a,c) wide, flattened, each composed of 7 pointed lobes: A, B, C, X, E2, E1b, E1a (respectively from the inner to the outer); A much shorter than B; pygidium (Figs 6a, 7a) protruding, medioventrally positioned between the urogomphal insertions.

### Eustrachinensis, second and third instar larvae

General morphology very similar to that described above for the first instar, except for: progressive increasing of relative dimensions (see Table 1); presence of secondary setae on antennomere II (2 setae); sensorial appendage much shorter than antennomere IV; retinaculum progressively longer and more falcate; stipe with wider and sharp sub-basal protuberance; labial palpomere I wider than II; tibia subequal to tarsus; pronotum about as wide as meso- and metanotum; dorsal plates of terminal disk slightly longer; lobes of urogomphi relatively longer and more slender; lobe E1a slightly longer than E1b.

**Table 1.** Measurements (mm) of three instars (L1, L2, L3) of Eustra chinensis and the third instar (L3) of Eustra sp.Taiwan. BL = body length (from tip of mandibles to the apex of terminal disk); HW = cephalic capsule maximum width (at the base of the antennae); HL = cephalic capsule medial length (mesodorsally, from occipital foramen to anterior margin of frontoclypeolabrum); PW = prothorax maximum width; PL = prothorax medial length; TDW = terminal disk maximum width (at the level of lateral plates); DPL = dorsal plates length (from base, near articulation, to the medial apex).

	Eustra chinensis L1	Eustra chinensis L2	Eustra chinensis L3	Eustra sp. L3
BL	1.75	2.7	3.02	-
HW	0.39	0.5	0.68	-
HL	0.21	0.28	0.37	-
PW	0.32	0.45	0.65	0.8
PL	0.19	0.28	0.42	0.55
TDW	0.5	0.7	0.98	1.42
DPL	0.22	0.33	0.5	0.62

### Eustra sp. Taiwan, third instar larva

Unfortunately, the specimen is damaged and portions of its head and legs are missing. Only basal part of head capsule, basal half of a mandible, thorax, basal part of legs, entire abdomen and terminal disk are intact. However, there is one low-resolution image of the entire specimen, which provides only limited information of some structural details.

General larval structure and most characters of the terminal disk (Fig. 6b) are very similar to those described above, especially as compared with the third instar of Eustra chinensis, except for the following minor differences:

(1) lobe A of urogomphi about as long as B (A much shorter than B in Eustra chinensis); (2) E1a thinner and more elongate than E1b (subequal or only slightly longer to E1b in Eustra chinensis); (3) lobes A very close medially, almost touching (distinctly separated medially in Eustra chinensis).

## Discussion

Eustra larvae are highly modified compared with the other known larvae of Ozaenini, and have several unique structures that make their identification easy. These include:

1. Antennae 3-jointed (Figs 1c, 2c). Paussinae larvae generally show 4-jointed antennae, a condition typical for adephagans. The reduction to 3 joints in Eustra is clearly due to the fusion of antennomeres II and III.

2. Mandible apically bidentate with sub-basal retinaculum (Figs 1d, 3b). A bidentate mandibular apex is also present in all known myrmecophilous Paussini larvae except Arthropterus, but in this tribe the second tooth is thought to be a subapically displaced retinaculum (Di Giulio and Moore 2004). In addition to the bidentate apex (Fig. 3b), a small subtriangular and basally directed retinaculum is present in Eustra first instars, and it becomes longer and more falcate in later instars.

3. Ental margin of retinaculum with additional small sub-basal tooth (Fig. 1d). This margin is straight only in Physea, while it is more or less sinuate (basal half convex, distal half concave) in all other known ozaenine genera. The presence of a sub-basal tooth on the ental margin in Eustra may be an adaptation for piercing and holding their prey.

4. Maxillary palp 3-jointed (Fig. 1e). The reduction of the palpomeres from 4 to 3 is a common feature of known Paussini larvae except for Platyrhopalopsis and Arthropterus. In the genus Paussus the reduction is due to the fusion of basal palpomere with the stipe. In Eustra the basal palpomere is only partially fused with stipe but still recognizable, and the actual reduction is due to the fusion of palpomeres II+III.

5. Galea extremely long and apically sharp (Figs 1e, 3c). The galea of Eustra is two-jointed as is typical of ozaenines but it is highly modified: it is very strong, up-curved, and almost two times longer than the maxillary palp and almost three times longer than the lacinia. The apex is hook-like and unusually sharp, which would provide an effective tool for capturing and holding prey.

6. Strongly asymmetric tarsal claws (Figs 4b, 5b). All Metriini, Mystropomini, and Ozaenini larvae have legs with two tarsal claws of unequal size, the anterior distinctly longer than posterior, while myrmecophilous Paussini larvae have only a single claw (presumably the anterior). In Eustra the posterior tarsal claw is extremely small and almost obsolescent.

7. Hypopleurite VI with ventrolateral, elongate digitiform protuberance, tipped by strong spine-like seta (Figs 4d, 5d). This peculiar sensorial structure is unique to the genus Eustra.

8. Most sternal and pleural setae of the abdomen elongate and clavate at apex (Figs 4c-d, 5c-d). Clavate sensilla have been described in the myrmecophilous genus Arthropterus (sensilla S-VIII possibly homolog to S-II, see Di Giulio and Moore 2004), which surround the terminal disk, and are also present on the thorax and cephalic capsule. In Eustra, a clavate modification affects most abdominal mechanoreceptors as well as most sensilla of the terminal disk (see below). In particular, the terminal disk has two types of sensilla, often alternate (i.e. dorsal plates): type 1 is very long, stick-like, and pointed at the apex; type 2 is short and clavate at the tip.

9. Lateral plates of terminal disk transverse, subtriangular and pointed at apex (Figs 6a-b, 7d), with straight margins. The lateral plates of the Metriini, Mystropomini and other Ozaenini are wide and broadly rounded. Lateral plates of Goniotropis (Ozaenini) (Fig. 6c) are transverse and widely separated from dorsal plates similar to those of Eustra.

10. Urogomphi flattened, wider and longer than dorsal plates, composed by 7 short triangular lobes (Figs 6a-b, 7a), acute or bidentate at apex, separated by V-shaped notches of different depths, very shallowas compared to other Ozaenini. The flattening and widening of the urogomphi and the reduction (Physea, Ozaenini) or absence (all Paussini, see for example Fig. 6d) of branches is a typical feature of myrmecophilous larvae (Di Giulio et al. 2003; Di Giulio and Moore 2004).

11. Absence of urogomphal lobe D and presence of the additional urogomphal lobe X (Figs 6a-b, 7a). The lobes of urogomphi were coded first in Metrius by Bousquet (1986) and his notation was later slightly modified for ozaenines to include the partial or total bipartition of lobes B (B1+B2) and E (E1+E2) (Vigna Taglianti et al. 1998). This notation works for all described ozaenine larvae except for Eustra, which do not have a lobe D, but rather have an additional lobe (here named “lobe X”) located between lobes C and E2. Lobe X may be interpreted as: (1) a unique lobe (X = F); (2) D-lobe distally displaced to the margin (X = D); or (3) an additional subdivision of lobe E (X = E3). Eustra larvae also have a unique subdivision of E1 (E1a, E1b).

12. Peculiar inflated sensilla S-I (Figs 7a,c,e) of different lengths present on surface of plates and urogomphi. Inflated sensilla S-I have been described in larvae of Platyrhopalopsis (Paussini) and Physea (Ozaenini) and have been considered as an adaptation to the myrmecophilous lifestyle (Di Giulio et al. 2003). The sensilla S-I of Eustra are very different from the homologous structures of the aforementioned taxa since these are mushroom-shaped, composed of an elongate basal stem, which emerges from a cuticular protuberance, and an apical irregular inflation.

Like in the other ozaenine genera, larvae of Eustra live in galleries that they dig in humid soil or rotten wood and close off with their terminal disk, which they use to trap prey. However, the larvae of Eustra are so specialized and modified that it is not possible to find clear synapomorphies with larvae of any of the other known ozaenine genera. Some of the peculiar adaptations discussed above are similar to, but not necessary homologous to, characteristics described for the myrmecophilous larvae of Paussini and Physea (See Table 2). Since the Eustra larva from Taiwan was found inside a nest of Pachycondyla javana,it is possible that some of these traits are adaptations to a myrmecophilous lifestyle. However, we think that it is more likely that these minute larvae feed on very small invertebrates like collembolans and Drosophila, which first instar ozaenine larvae consistently consume in the lab (Moore and Di Giulio, pers. obs.), than it is that they feed on Pachycondlya, which are relatively large-bodied ants. Instead, many of the unusual characters observed in these larvae could facilitate feeding on fast moving prey, including the very long radial mechanoreceptors (sensilla S-II) of the terminal disk which would sense the approach of fast collembolans, and modified mouthparts including the bidentate mandibular apex, second tooth of retinaculum, and hook-like galea which would help the larva hold onto motile prey. Other characters, such as the flattening and widening of the urogomphi, could be related to the miniaturization of the larval body. In the future, we hope to discover the larvae of the genus Dhanya, and compare its morphological structures with Eustra since they are hypothesized to be sister genera (Jeannel 1946; Stork 1985; Deuve 2001), as well as larvae of the species formerly classified in the genus Ozaenaphenops to search for support of its synonymy with Eustra (Deuve 2001).

**Table 2.** Characteristics of Eustra larvae that are similar to those found in mymecophilous larvae.

Eustra+Physea	
	galea elongate (but in a completely different way: in Physea galeomere I long, II short and truncate at apex; in Eustra I short and II very long and sharp)
	lacinia reduced
	prementum elongate and tapered from base to apex
	labral spine wide
	ligula absent
	urogomphal lobes partially fused
	head short and transverse
	frontoclypeolabrale wide and transverse
	coronal suture short
	anterior arms of frontal sutures only slightly sinuate
	stemmata absent
	retinaculum in first instar triangular, inward directed
	sensilla S-I inflated
Eustra+Paussini	
	mandibles apically bidentate
	number of maxillary palp articles reduced (but in a completely different way, see Discussion)
	second tarsal claw reduced (in Paussini second claw is absent)
	sensilla clavate or inflated (only in Arthropterus clavate sensilla S-II, inflated S-I in Platyrhopalopsis)
	urogomphal lobes short rather then strongly branched
	urogomphi flat and wide
	antennae short and strongly directed medially
	stemmata absent
	sensorial appendage elongate
	head shortened and distinctly transverse

## References

[B1] Ball GE McCleve S (1990) The Middle American genera of the tribe Ozaenini with notes about the species in southwestern United States and selected species from Mexico. Quaestiones Entomologicae 26:30-116

[B2] Beutel RG (1992) Study on the systematic position of Metriini based on characters of the larval head (Coleoptera: Carabidae). Systematic Entomology 17:207-218

[B3] Bousquet Y (1986) Description of first-instar larva of *Metrius contractus* (Coleoptera: Carabidae) with remarks about phylogenetic relationships and ranking of the genus *Metrius*. The Canadian Entomologist 118:373-388

[B4] Bousquet Y Goulet A (1984) Notation of primary setae and pores on larvae of Carabidae (Coleoptera: Adephaga). Canadian Journal of Zoology 62:573-588

[B5] Costa C Vanin SA Casari-Chen SA (1988) Larvas de Coleptera do Brasil. Museu de Zoologia, Sao Paulo: Universidade de Sao Paulo, 282 pp. + 165 pls.

[B6] Deuve T (2001) Le genre *Eustra* Schmidt-Goebel, 1846, insectes (Coleoptera, Paussidae, Ozaeninae) à genitalia femelles orthotopiques. Zoosystema 23:547-578

[B7] Di Giulio A Fausto AM Taddei AR Vigna Taglianti A (2000) The terminal disk of *Pachyteles* larvae (Coleoptera, Carabidae, Paussinae): a morphological study. In: Brandmayr P Lövei G Zetto Brandmayr T Casale A Vigna Taglianti A (Eds) Natural History and Applied Ecology of Carabid Beetles, Proceedings of the IX European Carabidologists Meeting (26–31 July, 1998, Camigliatello, Cosenza, Italy). Pensoft, Sofia-Moscow, 89–93

[B8] Di Giulio A Moore W (2004) The first-instar larva of the genus *Arthropterus* (Coleoptera: Carabidae: Paussinae): implications for evolution of myrmecophily and phylogenetic relationships within the subfamily. Invertebrate Systematics 18:101-115

[B9] Di Giulio A Vigna Taglianti A (2001) Biological observations on *Pachyteles* larvae (Coleoptera: Carabidae: Paussinae). Tropical Zoology 14:157-173

[B10] Di Giulio A Fattorini S Kaupp A Vigna Taglianti A Nagel P (2003) Review of competing hypotheses of phylogenetic relationships of *Paussinae* (Coleoptera: Carabidae) based on larval characters. Systematic Entomology 28:509-537

[B11] Di Giulio A Maurizi E Hlaváč P Moore W (2011) The long-awaited first instar larva of *Paussus favieri* (Coleoptera: Carabidae: Paussini). European Journal of Entomology 108:127-138

[B12] Eidmann H (1937) Die Gäste und Gastverhältnisse der Blattschneiderameise Atta sexdens L. Zeitschrift fuer Morphologie und Oekologie der Tiere 32:391-462

[B13] Geiselhardt SF Peschke K Nagel P (2007) A review of myrmecophily in ant nest beetles (Coleoptera: Carabidae: Paussinae): linking early observations with recent findings. Naturwissenschaften 94:871-894 17563864 10.1007/s00114-007-0271-x

[B14] Harris RA (1979) A glossary of surface sculpturing. Occasional Papers in Entomology 28:1-31

[B15] Jeannel R (1946) Coléoptères Carabiques de la Région Malgache (première partie). Faune de l’Empire français 6:1-372

[B16] Lawrence JF (1991) Order Coleoptera. In: Stehr FW (Ed) Immature insects, Vol. 2, Kendall/Hunt Publishing Company, Dubuque, Iowa, 144–298

[B17] Luna de Carvalho E (1989) Essay monographique des Coléoptèteres Protopaussines et Paussines. Memorias do Institudo de Investigaçao Científica Tropical, (segunda série), Lisboa, 70, (1987), 1028 pp.

[B18] Moore W (2006) Molecular phylogenetics, systematics, and natural history of the flanged bombardier beetles (Coleoptera: Adephaga: Carabidae: Paussinae). PhD thesis, Tucson, Arizona, USA: The University of Arizona

[B19] Moore W (2008) Phylogeny of the Western Hemisphere Ozaenini (Coleoptera: Carabidae: Paussinae) based in DNA sequence data. Annals of the Carnegie Museum 77:79-92

[B20] Moore W Di Giulio A (2006) Description and behaviour of *Goniotropis kuntzeni* larvae (Coleoptera: Carabidae: Paussinae: Ozaenini) and a key to genera of Paussinae larvae. Zootaxa 111:1-19

[B21] Moore W Di Giulio A (2008) *Metrius* Eschscholtz (Carabidae: Paussinae) is not a millipede specialist. The Pan-Pacific Entomologist 84:33-34

[B22] Moore W Di Giulio A Song X (2010) Paussinae (Coleoptera: Carabidae) larvae recently discovered in Asia and Madagascar. In: Fikacek M, Skuhrovec J, Sipek P (Eds) Abstracts of the Immature Beetles Meeting 2009, October 1 2, Prague, Czech Republic. Acta Entomologica Musei Nationalis Pragae 50:323-342

[B23] Oberprieler RG (1985) Paussidae. In: Scholtz CH Holm E (Eds) Insects of Southern Africa, Butterworth Publishers (Pty) Ltd., Durban, 196–198

[B24] Stork NE (1985) *Dhanya*, a South-east Asian genus of ozaenine ground beetles. Journal of Natural History 19:1113-1138

[B25] Vigna Taglianti A Santarelli F Di Giulio A Oliverio M (1998) Phylogenetic implications of larval morphology in the tribe Ozaenini (Coleoptera, Carabidae). In: Ball GE Casale A Vigna Taglianti A (Eds) Phylogeny and classification of Caraboidea (Coleoptera: Adephaga), Proceedings of a Symposium (28 August, 1996, Florence, Italy), XX International Congress of Entomology, Museo Regionale di Scienze Naturali, Torino, Atti, 273–296

